# Performance Enhancement of SPR Biosensor Using Graphene–MoS_2_ Hybrid Structure

**DOI:** 10.3390/nano12132219

**Published:** 2022-06-28

**Authors:** Haoyuan Cai, Mengwei Wang, Zhuohui Wu, Jing Liu, Xiaoping Wang

**Affiliations:** 1 Ocean College, Zhejiang University, Zhoushan 316021, China; hycai@zju.edu.cn (H.C.); wmw@zju.edu.cn (M.W.); zhuohui_wu@zju.edu.cn (Z.W.); 2 Key Laboratory of Ocean Observation-Imaging Testbed of Zhejiang Province, Zhejiang University, Zhoushan 316021, China; 3 The Engineering Research Center of Oceanic Sensing Technology and Equipment, Ministry of Education, Zhoushan 316021, China; 4 School of Information Engineering, Jimei University, Xiamen 361021, China

**Keywords:** biosensor, MoS_2_, graphene, SPR sensor, high sensitivity

## Abstract

We investigate a high-sensitivity surface plasmon resonance (SPR) biosensor consisting of a Au layer, four-layer MoS_2_, and monolayer graphene. The numerical simulations, by the transfer matrix method (TMM), demonstrate the sensor has a maximum sensitivity of 282°/RIU, which is approximately 2 times greater than the conventional Au-based SPR sensor. The finite difference time domain (FDTD) indicates that the presence of MoS_2_ film generates a strong surface electric field and enhances the sensitivity of the proposed SPR sensor. In addition, the influence of the number of MoS_2_ layers on the sensitivity of the proposed sensor is investigated by simulations and experiments. In the experiment, MoS_2_ and graphene films are transferred on the Au-based substrate by the PMMA-based wet transfer method, and the fabricated samples are characterized by Raman spectroscopy. Furthermore, the fabricated sensors with the Kretschmann configuration are used to detect okadaic acid (OA). The okadaic acid–bovine serum albumin bioconjugate (OA-BSA) is immobilized on the graphene layer of the sensors to develop a competitive inhibition immunoassay. The results show that the sensor has a very low limit of detection (LOD) of 1.18 ng/mL for OA, which is about 22.6 times lower than that of a conventional Au biosensor. We believe that such a high-sensitivity SPR biosensor has potential applications for clinical diagnosis and immunoassays.

## 1. Introduction

Surface plasmon resonance (SPR) is one of the most powerful optical-sensing technologies due to its high sensitivity, real-time, and label-free detection [1,2,3,4,5,6]. SPR-based biosensors have many practical applications, such as medical diagnostics, food safety, virus detection, etc., as they can be coupled with various molecular recognition elements, either chemical receptors (nanomaterials, molecularly imprinted polymers (MIPs), etc.) or bioreceptors (enzymes, nucleic acids, antibodies, etc.) [7,8,9,10]. In general, Ag and Au are widely used as plasmonic materials for SPR sensors. Au film is preferred because of its excellent resistance to corrosion and oxidation in different external environments [11]. However, bioreceptors are poorly immobilized on the surface of Au film, limiting the sensitivity of the Au-based SPR biosensor.

Many chemical methods are used to enhance the immobilization of bioreceptors. Among them, the self-assembly monolayer (SAM) has been widely proven to be particularly effective because of its simplicity of fabrication, reproducibility, and good temperature stability [12,13,14]. Many studies have reported using SAMs to improve the sensitivity of SPR sensors. Taylor et al. [13] report a limit of detection (LOD) of 0.3 ng/mL for Tetrodotoxin (TTX) by using a mixed SAM layer. Kawaguchi et al. [14] employ the PEG-based SAM layer for detecting TNT with a LOD of 0.008 ng/mL.

In addition to these chemical methods, many biosensors, based on metallic nanoslits [15] or nanoholes structures [16,17], are proposed to enhance the sensitivity of the SPR sensors. However, the fabrication processes of these structures often involve complicated steps or require expensive, time-consuming electron beam lithography. Moreover, large-area fabrication is challenging.

To solve this problem, a large-area thin graphene layer is used to cover the Au film surface to improve the sensitivity of the SPR sensor [18,19]. Graphene is a monoatom thin planar sheet of sp_2_ carbon atoms well organized in the form of a honeycomb lattice. It provides better support for biomolecule adsorption due to its rich π conjugation structure and large surface area, making it a suitable dielectric layer for SPR sensing [20,21]. For example, Verma et al. [22] exploit graphene and silicon to improve the sensitivity of SPR biosensors. Wu et al. [23] employ 10-layer graphene to improve sensitivity, and the performance can be nearly 25% enhanced. At a certain wavelength range, more graphene layers result in higher sensor sensitivity. However, due to the large imaginary part of graphene, too many graphene layers will produce excessive an amount of damping in plasmonic waves, resulting in reduced detection accuracy.

In addition to graphene, other 2D materials, such as molybdenum disulfide (MoS_2_), have gained much attention for sensing applications. Monolayer MoS_2_ has many unique advantages, such as higher light absorption efficiency (5%), large direct bandgap (1.8 eV), and larger work function (5.1 eV), so it is widely used in the field of SPR sensing [24,25,26]. For example, Xue et al. [27] design a high-sensitivity SPR sensor by coating seven layers of MoS_2_ on the surface of a sensor. They demonstrate that the highest sensitivity is about 190°/RIU and the LOD of Hg^2+^ for the sensor is 1.0 pM. In addition, the emergence of the chemical vapor deposition (CVD) technique makes the large-area growth of MoS_2_ possible, which further facilitates the development of MoS_2_-based SPR sensors [28].

In this work, we propose a high-sensitivity SPR biosensor based on the graphene–MoS_2_ structure. MoS_2_ films are used to absorb more light energy and the monolayer graphene is used as the biomolecular recognition element due to its large surface area. Theoretical optimization, based on the transfer matrix method (TMM), shows that a maximum sensitivity ~282°/RIU is achieved, when the Au-based substrate is modified with four-layer MoS_2_ and monolayer graphene. The mechanism of sensitivity enhancement is discussed and explained theoretically. In the experiment, a PMMA-based wet transfer method is used to transfer MoS_2_ and graphene film on the Au-based substrate and the high-refractive-index (RI) sensitivity of the fabricated sensor is verified. In addition, the proposed biosensor is used to detect okadaic acid (OA) by the indirect competitive inhibition method. The experimental results demonstrate that the graphene–MoS_2_ hybrid structure can greatly reduce the LOD of the SPR biosensor.

## 2. Numerical Simulation

### Design the Proposed SPR Biosensor

**Figure 1:**
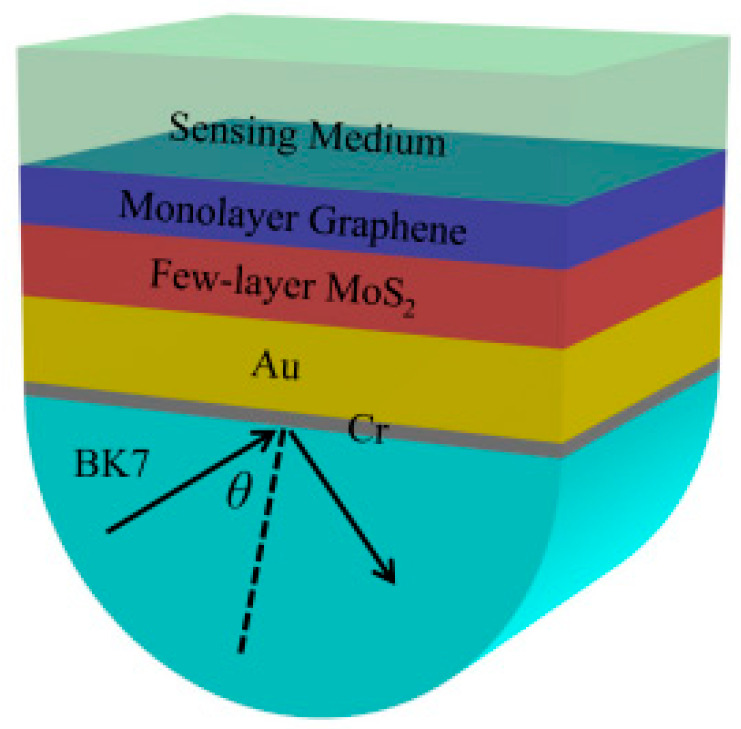
Schematic illustration of the designed SPR sensor.

In Figure 1, the designed SPR sensor is based on a prism, Cr layer, Au layer, few-layer MoS_2_, and monolayer graphene. In this structure, BK7 glass is used as a coupling prism and Au is used as a plasmonic material to excite the SPR effect. Few-layer MoS_2_ films are used to absorb more light energy and the monolayer graphene is used as the biological recognition component, which further improves sensor sensitivity. In the simulation, the thickness of the Cr layer is 5 nm and the thickness of the Au layer is 50 nm. The wavelength of the incident light source is 632.8 nm. At this wavelength, the RI of BK7 glass is 1.516 [29]. The RI of Au is obtained from the Drude–Lorentz model [30]. The RI of graphene is 3 + 1.1487i [31] and its thickness can be expressed as d_G_ = L∗0.34 nm, where L is the number of graphene layers. The RI of the MoS_2_ layer is 5.9 + 0.8i [32] and its thickness can be denoted as d_M_ = M∗0.65 nm, where M is the number of MoS_2_ layers.

**Figure 2:**
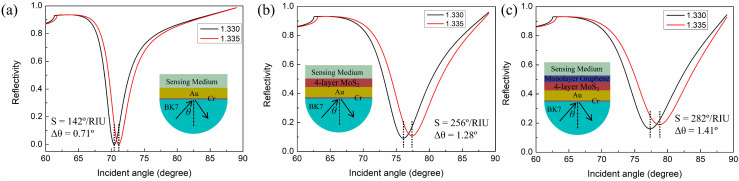
Reflectivity curves change with incident angles for (**a**) conventional Au-based sensor, (**b**) MoS_2_-based sensor, (**c**) graphene–MoS_2_ hybrid-structure sensor.

To evaluate sensing performance, we compare the RI sensitivities of three different structures: the conventional Au-based sensor, the MoS_2_-based sensor, and the graphene–MoS_2_ hybrid-structure sensor. TMM is used to calculate the reflectivity curves of various structures and the calculated results are shown in Figure 2. The sensitivity calculation formula of the SPR sensor is S=Δθ/Δn, where Δθ is the variation in resonance angle and Δn is the variation in the RI of the sensing medium. Figure 2a is the reflectivity curves of the conventional Au-based sensor, and the thickness of the Au film is 50 nm. From this figure, the resonance angle increases ∼0.71° when n_s_ changes from 1.330 to 1.335. Thus, the sensitivity of the Au-based sensor is 142°/RIU. In Figure 2b, we use the four-layer MoS_2_ to enhance the sensitivity and the sensitivity is calculated to be 256°/RIU. The high sensitivity is because the presence of the MoS_2_ layer increases the efficiency of light absorption of the sensor. Most of the incident light energy is transferred to free electrons on the sensor surface, so more surface plasmons are generated, which results in the higher sensitivity [33,34]. In Figure 2c, the monolayer graphene is covered on top of the MoS_2_ layer to further improve the sensitivity. The resonance angle shifts by ∼1.41° and the sensitivity increases to 282°/RIU. Obviously, the monolayer graphene does not significantly enhance the RI sensitivity of the proposed sensor. This is due to the fact that the light absorption efficiency of graphene is lower than that of MoS_2_. The primary function of the monolayer graphene is used as a biomolecular recognition component. In biological experiments, the graphene provides a large surface area for adsorbing biomolecules, which lowers the LOD of the sensor [35].

**Figure 3:**
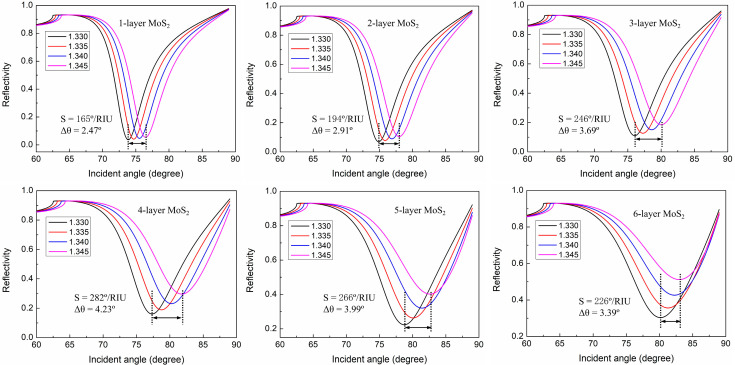
Reflectivity curves change with incident angles for graphene–MoS_2_ hybrid-structure sensor in different sensing medium, where the number of MoS_2_ layers increases from M = 1 to M = 6.

To study the influence of the number of MoS_2_ layers on the sensitivity of the graphene–MoS_2_ hybrid-structure sensor, we calculate the reflectivity curves in a different sensing medium, as shown in Figure 3. According to Figure 3, the SPR curves become wider and the resonance angle obviously shifts to higher angle values with the increase in the MoS_2_ layers. This is due to the relatively high RI in the real parts of the MoS_2_ material, which produces excessive amount of damping in the plasmonic wave [36]. Furthermore, we can see that the sensitivity first increases and then decreases with the increase in MoS_2_ layers. This can be explained that as the number of MoS_2_ layers continuously increases, the resonance angle will increase to 90°, but the detection angle cannot reach 90°, limiting the sensitivity of the SPR sensor [37]. When the number of MoS_2_ layers is four (M = 4), the highest sensitivity of 282°/RIU is obtained, which is significantly improved as compared to the other reported works [22,23,27].

**Figure 4:**
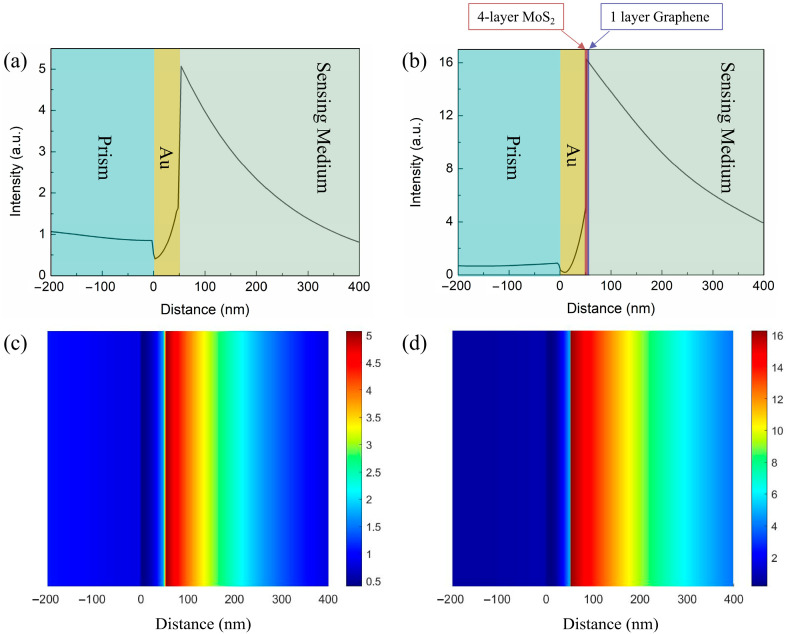
The electric field distributions for (**a**,**c**) conventional Au-based sensor and (**b**,**d**) graphene–MoS_2_ hybrid-structure sensor.

In order to clearly demonstrate the electric field enhancement of the proposed sensor, we compare the electric field distribution in two configurations of the conventional Au-based sensor and the graphene–MoS_2_ hybrid-structure sensor with four-layer MoS_2_ at resonance condition. The finite difference time domain (FDTD) method is used to simulate the electric field distribution and the calculated results are shown in Figure 4. Compared with the Au-based sensor, the electric field of the proposed sensor in Figure 4b can have an evident improvement by coating the MoS_2_ and graphene film. On one hand, the graphene–MoS_2_ hybrid structure improves the absorption of light energy. On the other hand, the absorbed light energy is transferred to the free electrons, resulting in strong coupling on the graphene surface, ultimately enhancing the surface electric field. The electric-field-enhanced region has a more sensitive response to the slight variation in RI of the sensing medium.

## 3. Experiment

### 3.1. Materials and Reagents

NaCl, H_2_SO_4_, HCl, NaOH, H_2_O_2_, anisole, KOH, acetone, and poly (methyl methacrylate) (PMMA, molecular weight ≥20,000) were obtained from Sinopharm Chemical Reagent Co., Ltd. (Shanghai, China). N-hydroxysuccinimide (NHS), 1-ethyl-3-(3-dimethylaminopropyl)-carbodiimide hydrochloride (EDC), ethanolamine and phosphate-buffered saline (PBS) were purchased from Shanghai Aladdin Biochemical Technology Co., Ltd. Okadaic acid (OA) with >95% purity (HPLC-grade), the okadaic acid–bovine serum albumin bioconjugate (OA-BSA), and anti-OA monoclonal antibody (OA-mAb) were purchased from Anti Biological Technology Co., Ltd. (Shenzhen, China). A 0.2 M NaOH solution was used as the regenerant. All reagents and solvents are analytical grade and were used without further purification. Deionized water (18.2 MΩ·cm) was used throughout the work.

### 3.2. Sample Fabrication

#### 3.2.1. The Fabrication of the Conventional Au-Based Sensor

**Figure 5:**
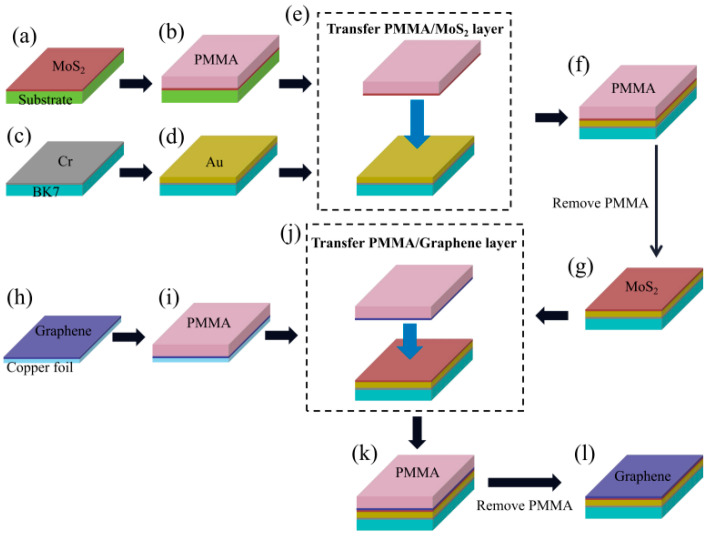
Schematic diagram of fabrication processes of the graphene–MoS_2_ hybrid-structure sensor. (**a**) MoS_2_ deposition using CVD, (**b**) PMMA spin-coating, (**c**) Cr deposition using magnetron sputtering, (**d**) Au deposition using magnetron sputtering, (**e**) separation of PMMA/MoS_2_ from SiO_2_/Si substrate using KOH, (**f**) PMMA/MoS_2_ transferring to Au-based substrate, (**g**) PMMA removal using acetone, (**h**) monolayer-graphene deposition using CVD, (**i**) PMMA spin-coating, (**j**) etching the Cu foil using FeCl_3_, (**k**) PMMA/graphene transferring to MoS_2_-based substrate, (**l**) PMMA removal using acetone.

**Figure 6:**
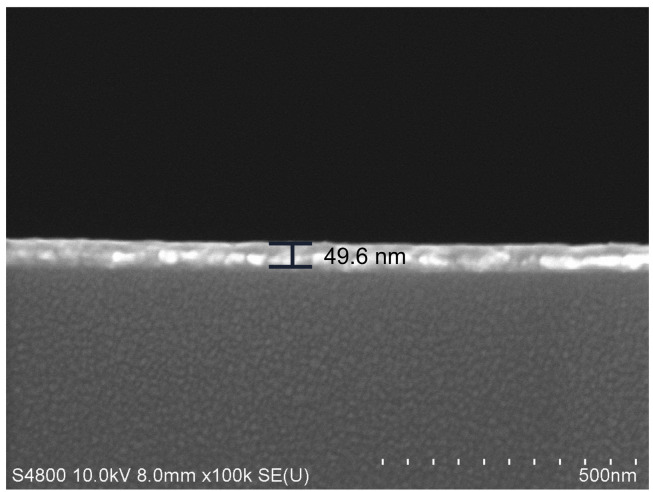
The SEM images of the conventional Au-based sensor.

The conventional Au-based sensor and graphene–MoS_2_ hybrid-structure sensor with different numbers of MoS_2_ layers are fabricated in this paper and the fabrication processes are shown in Figure 5. First, a 5 nm Cr layer and 50 nm-thick Au layer were sequentially deposited on the polished BK7 glass substrate by magnetron sputtering, where the Cr layer was used as an adhesion layer (See Figure 5c,d). The SEM image of Au-based sensor is shown in Figure 6. We can see that the thickness of the Au film is 49.6 nm.

#### 3.2.2. The Transfer Process of MoS_2_ Film

Continuous 2~6-layer MoS_2_ film purchased from SixCarbon Technology was grown on SiO_2_/Si substrate by CVD technique. The few-layer MoS_2_ films were transferred onto the Au-based substrate by a PMMA-based wet transfer method [38]. In the MoS_2_ transfer process, first, MoS_2_ grown on SiO_2_/Si substrate was spin-coated with PMMA (4% in anisole) at 3000 rpm for 1 min and baked at 120 C for 3 min. Then, samples with the PMMA coating were then soaked in a 2 mol/L KOH solution for 2 h, in which the PMMA/MoS_2_ layer was separated from the Si substrate due to etching of SiO_2_. Next, the PMMA/MoS_2_ layers were washed in DI water, scooped using an Au-based substrate, dried under ambient conditions, and baked at 80 °C for 1 h. Finally, the acetone was used to dissolve the PMMA. We can complete the transfer of MoS_2_ to obtain the MoS_2_-based substrate (See Figure 5a–g).

#### 3.2.3. The Transfer Process of Graphene Film

The monolayer graphene purchased from SixCarbon Technology was synthesized on Cu foil using the CVD technique. Similar to the transfer method of the MoS_2_ layer, we transferred the monolayer graphene on MoS_2_-based substrate to obtain the proposed biosensor. First, a thin layer of PMMA was spin-coated onto the surface of graphene/Cu foil at 500 rpm for 10 s, followed by 20 s at 2500 rpm. Then, the PMMA/graphene/Cu foil was baked at 120 °C for 3 min; then, the Cu foil was etched in a 1 mol/L FeCl_3_ solution for 1 h. After the Cu foil was dissolved, the PMMA–graphene film was repeatedly washed with sufficient DI water. Next, the PMMA/graphene sample was carefully transferred onto the surface of MoS_2_-based substrate. Then, the sample was dried at 90 °C for 60 min to enable the PMMA–graphene layer to firmly adhere to the substrate. Finally, the acetone was used to dissolve the PMMA and the fabricated samples were thoroughly washed with DI water (See Figure 5h–l). Through the above steps, we fabricated the graphene–MoS_2_ hybrid-structure sensor with different numbers of MoS_2_ layers.

**Figure 7:**
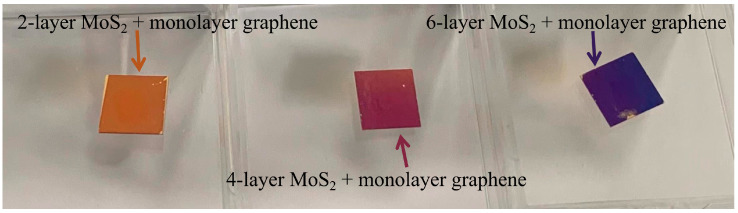
Photograph of the fabricated graphene–MoS_2_ hybrid-structure sensor with different layers of MoS_2_ and monolayer graphene.

**Figure 8:**
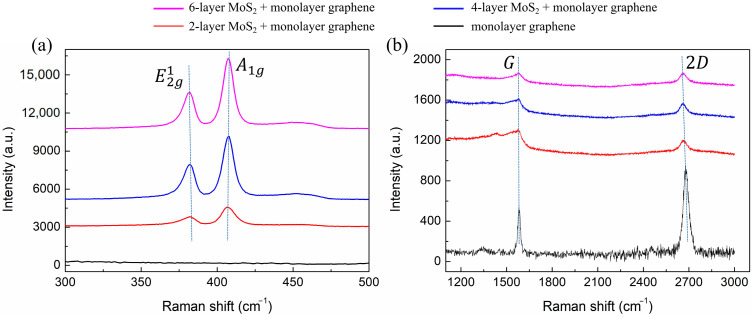
Raman spectra of monolayer graphene on SiO_2_ substrate and the fabricated graphene–MoS_2_ hybrid-structure sensor with different layers of MoS_2_ for (**a**) 300~500 cm^−^^1^; (**b**) 1100~3100 cm^−^^1^.

The photograph of the fabricated graphene–MoS_2_ hybrid-structure sensor with different layers of MoS_2_ and monolayer graphene is shown in Figure 7. We can see the different colors correspond to the different numbers of MoS_2_ layers. Raman spectra of fabricated samples are characterized by EnSpectrR532 at a laser wavelength of 532 nm and the corresponding spectra are shown in Figure 8. In the low-frequency region, two strong peaks $E_{2g}^{1}$ and $A_{1g}$, which locate at 382.9 cm^−1^ and 406.5 cm^−1^, respectively, are shown in Figure 8a. As the layer number increases from 2-layer to 6-layer, a slight red shift in the $E_{2g}^{1}$ band and a slight blue shift in the $A_{1g}$ band are observed. These findings suggest that MoS_2_ films are successfully transferred in graphene–MoS_2_ hybrid structure [39]. In the high-frequency band, the black line in Figure 8b indicates two characteristic peaks of graphene at 1581.2 cm^−1^ (G-band) and 2679.1 cm^−1^ (2D-band). According to previous reports, the number of graphene layers depends on the intensity ratios $I_{2D}/I_{G}$. When the $I_{2D}/I_{G}$ of this spectrum is approximately 2, it represents a typical spectrum of monolayer graphene [40]. In addition, for the red, blue, and pink lines, both the G and 2D peaks of graphene appear in the same location. These results indicate that monolayer graphene film was successfully transferred in a graphene–MoS_2_ hybrid structure.

### 3.3. RI-Sensing Experiments

**Figure 9:**
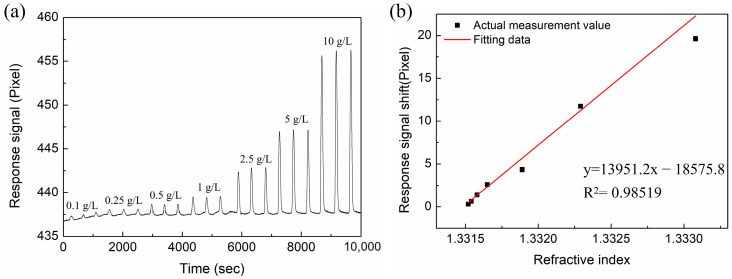
(**a**) The response curves of NaCl solution with different concentrations for the conventional Au-based biosensor. (**b**) The corresponding linear fitting line.

**Figure 10:**
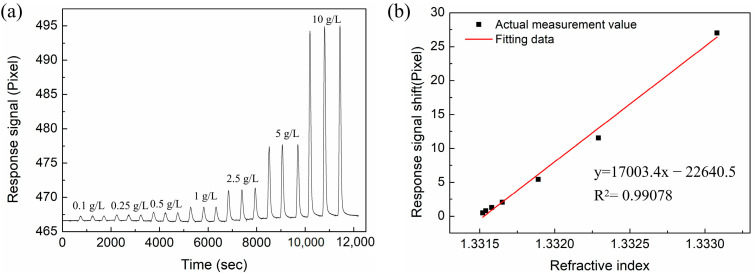
(**a**) The response curves of NaCl solution with different concentrations for the proposed biosensor based on two layers of MoS_2_. (**b**) The corresponding linear fitting line.

**Figure 11:**
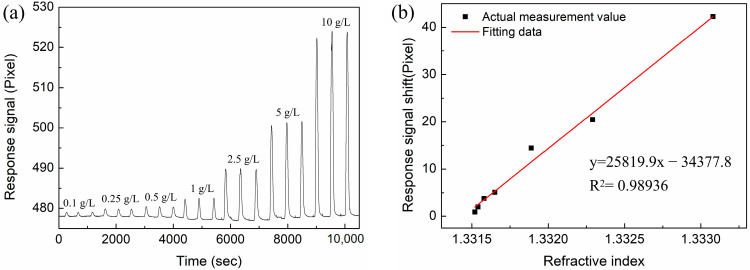
(**a**) The response curves of NaCl solution with different concentrations for the proposed biosensor based on four layers of MoS_2_. (**b**) The corresponding linear fitting line.

**Figure 12:**
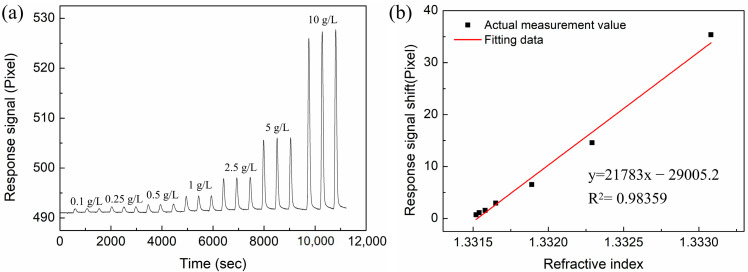
(**a**) The response curves of NaCl solution with different concentrations for the proposed biosensor based on six layers of MoS_2_. (**b**) The corresponding linear fitting line.

In this section, we compare the RI sensitivity of the Au-based sensor and the proposed sensor with different layers of MoS_2_. The Abbe refractive index meter was used to measure the RI of the NaCl solutions at room temperature (26 °C). The NaCl solution with concentration ranges of 0.1 g/L, 0.25 g/L, 0.5 g/L, 1 g/L, 2.5 g/L, 5 g/L, and 10 g/L were prepared, and their corresponding refractive indices are 1.33152, 1.33154, 1.33158, 1.33165, 1.33189, 1.33229, and 1.33308, respectively. In the experiment, deionized water was first injected into the sensor for a sufficient time to ensure the stability of the baseline. Then, the NaCl solution and deionized water were sequentially injected as a cycle, and the process was repeated to test different concentrations of NaCl solutions. Every concentration was measured three times, and the results were recorded in computer software. Figure 9 shows the response signal for the conventional Au-based biosensor in different RI solutions. From the linear fitting curve of Figure 9b, the RI sensitivity is 13,951.2 pixel/RIU with linearity of 0.98519. Similarly, NaCl solutions with different concentrations were injected into the sensing region of proposed sensor with different layers of MoS_2_. The response spectra are shown in Figure 10a, Figure 11a and Figure 12a. In Figure 10b, Figure 11b and Figure 12b, linear fittings are conducted to obtain the corresponding sensitivities. The sensitivity values are 17,003.4, 25,819.9, and 21,783.3 pixel/RIU for the proposed sensors with 2, 4, and 6 layers of MoS_2_, respectively, and all the correlation coefficients (R^2^) are higher than 0.98. When the number of MoS_2_ layers is four, the maximum sensitivity is obtained, which is 1.85 times that of conventional Au-based sensor. A good agreement is observed between the experimental results and the theoretical calculation results.

### 3.4. Okadaic Acid Detection Experiment

#### 3.4.1. Fabrication of SPR Immunosensor

The surface of graphene has abundant functional groups, which can be covalently bound to antigen or antibody molecules. For the pretreatment of the graphene-based SPR-sensing chip, the chip was rinsed thoroughly with plenty of water, and dried with nitrogen. Then, the carboxyl group of graphene was activated by NHS/EDC mixture solution (M:M = 4:1, v:v = 1:1) for 30 min, followed by washing in plenty of water. Afterwards, the OA-BSA conjugate (1 mg/mL) was dissolved in PBS buffer and was dropped onto the chip surface and incubated for 1 h. Finally, a 1 mol/L ethanolamine solution (pH 8.5) was used to block the nonspecific interaction on the biosensor surface.

#### 3.4.2. Determination of OA

**Figure 13:**
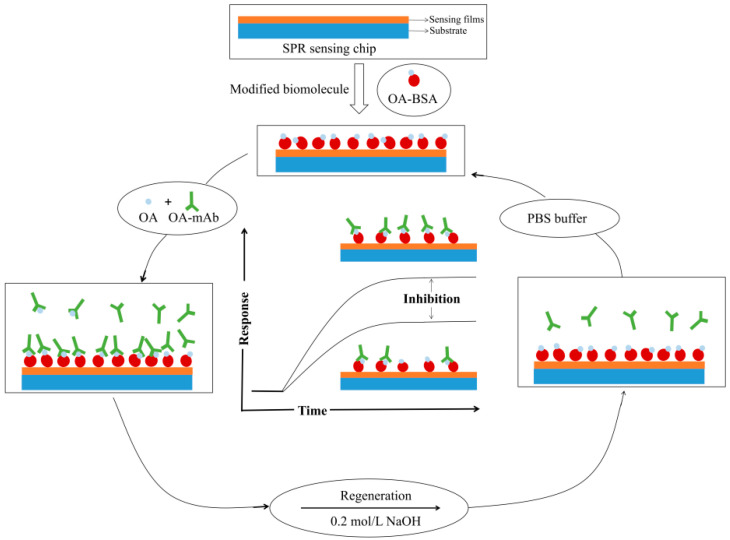
The schematic diagram of the detection process for OA.

**Figure 14:**
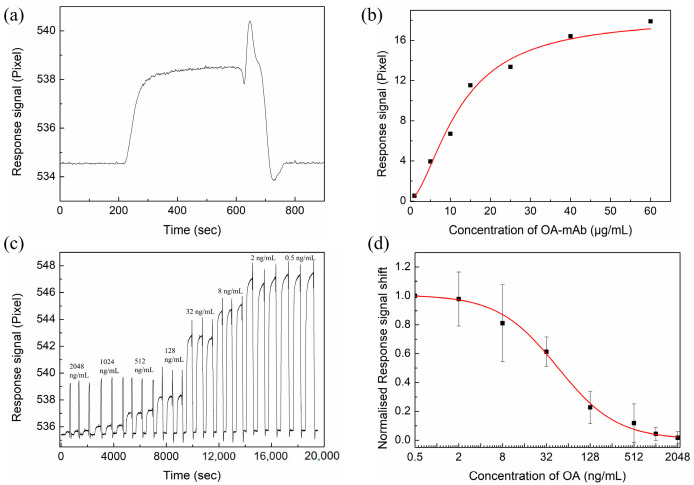
(**a**) The specific interaction process between OA-mAb (5 μg/mL) and the OA-BSA followed by regeneration step. (**b**) The shift in response signal with increasing concentration of OA-mAb. (**c**) The SPR response signal curves of OA solution at different concentrations for the proposed SPR biosensor. (**d**) Calibration curve for the detection of OA by indirect competitive inhibition.

The low-molecular-weight analyte OA was detected by the proposed SPR sensor with four-layer MoS_2_ by an indirect competition inhibition method [41,42], and the schematic diagram of the detection process for OA is shown in Figure 13. Figure 14a demonstrates the specific interaction process of OA-mAb and OA-BSA immobilized on the sensor chip surface. First, PBS buffer was injected over the sensor chip to obtain a stable baseline before the measurement. Then, the OA-mAb solution (5 μg/mL) was injected into the reactor. The free OA-mAb was captured by OA-BSA on the sensor chip surface and the response signal was increased. At the end of the detection cycle, the captured antibodies were removed by injecting NaOH (0.2 M) solution for the regeneration, and the response signal returned to the original baseline position.

For the indirect competition inhibition method, the concentration of antibody is a key parameter affecting sensitivity. To obtain the appropriate antibody concentration, a series of concentrations of OA-mAb (1~60 μg/mL) were injected into the sensor chip. Figure 14b shows the resonant pixel shifts with an increasing concentration of OA-mAb. The resonant pixel shift increases rapidly before the antibody concentration reaches 15 μg/mL, followed by a slower rate of increase. Therefore, a 15 μg/mL OA-mAb concentration was used in subsequent OA experiments.

Firstly, the OA standard solutions were incubated with the 15 μg/mL OA-mAb solution for 30 min. Subsequently, the equilibrated mixtures, containing a series of OA concentrations (0.5~2048 ng/mL) and 15 μg/mL OA-mAb, were injected into the sensor chip. The reproducibility of the immune reaction was assessed by analysis of each concentration of OA mixture solutions three times. The obtained SPR response signal curves are shown in Figure 14c. From Figure 14c, it can be seen that the shift in the resonant pixel decreases with the increase in the OA concentration in the solution. This is due to the OA in free solution inhibiting the binding interaction of OA-mAb with the OA-BSA immobilized on the sensor chip, resulting in a decrease in the response signal. The extent of reduction is directly proportional to the OA concentration. Figure 14d is the percentage of inhibition with respect to the OA concentration. It can be inferred from the sigmoidal calibration curve that the linear detection range of the proposed OA biosensor is from 4 ng/mL to 512 ng/mL. The LOD is estimated to be 1.18 ng/mL based on the LOD formula [43], which is approximately 22.6 times lower than the conventional Au-based sensor (26.7 ng/mL).

## 4. Conclusions

In this work, a high-sensitivity SPR biosensor composed of MoS_2_ and graphene is proposed, and its performance was investigated through a simulation and experiments. MoS_2_ films with a high light absorption efficiency were utilized to promote the transfer of electrons, which resulted in a significant enhancement of the electric field on the sensor’s surface. Monolayer graphene was employed as the biological recognition component to further increase the sensitivity of the sensors. The numerical results show that the maximum sensitivity ~282°/RIU is achieved, when the sensor is modified with four-layer MoS_2_ and monolayer graphene. In the experiment, the proposed sensor was used to measure the RI sensitivity and detect the OA concentration. The result of the RI experiments show that the RI sensitivity of the proposed sensor is approximately 1.85 times higher than the conventional Au sensor. In addition, the LOD of OA for the proposed sensor is 1.18 ng/mL, which is about 22.6 times lower than the conventional Au sensor. These results suggest that this device has the potential for clinical diagnostics and chemical detection.

## Data Availability

The data presented in this study are available on request from the corresponding author upon reasonable request.
